# The Role of Stress in Stable Patients with Takotsubo Syndrome—Does the Trigger Matter?

**DOI:** 10.3390/jcm11247304

**Published:** 2022-12-09

**Authors:** Gassan Moady, Otman Ali, Rania Sweid, Shaul Atar

**Affiliations:** 1 Department of Cardiology, Galilee Medical Center, Nahariya 2210001, Israel; 2 Azrieli Faculty of Medicine, Bar Ilan University, Safed 5290002, Israel; 3 Department of Internal Medicine, Galilee Medical Center, Nahariya 2210001, Israel; 4 Biostatistics Unit, Galilee Medical Center, Nahariya 2210001, Israel

**Keywords:** takotsubo, cardiomyopathy, trigger, stress, outcome

## Abstract

Background: Takotsubo syndrome (TTS) is a unique type of reversible cardiomyopathy that predominantly affects elderly women. The role of physical and emotional stress in the pathophysiology of TTS is well established. However, the association between preceding emotional triggers and clinical outcomes in stable patients has not yet been fully investigated. We aimed to investigate the association between emotional triggers before symptom onset and clinical outcomes in stable patients with TTS. Methods: This is a retrospective cohort study based on the data of patients with ICD-9 discharge diagnosis of TTS between 2017 and 2022. Patients were divided into two groups: with and without obvious emotional trigger before symptom onset. Demographic, laboratory, echocardiographic, and clinical outcomes were obtained and compared between the two groups. Results: We included 86 patients (93% were women, mean age 68.8 ± 12.3 years). Of them, 64 (74.4%) reported an emotional trigger before symptom onset. Patients with a previous emotional trigger had a longer hospital stay (4.3 + 2.0 days vs. 3.0 + 1.4, *p* = 0.002) with no difference in in-hospital complications (32.8% vs. 13.6%, *p* = 0.069), with no difference in 30-day mortality, readmissions, or recurrence rate between the groups. Conclusions: Patients with TTS related to an emotional trigger may represent a different population from patients without a preceding trigger by having more symptomatic disease and longer hospital stay, yet with no difference in the 30-day outcomes.

## 1. Introduction

Takotsubo syndrome (TTS), also known as stress cardiomyopathy or apical ballooning syndrome, is a unique type of reversible cardiac dysfunction mediated by various neurohormonal processes, often preceded by a physical or emotional trigger [1,2,3]. According to previous studies, about two thirds of patients report a physical or mental trigger before symptom onset [1,2,3]. Several echocardiographic patterns have been reported, with apical ballooning being the most common variant [4]. The presentation of TTS often mimics acute coronary syndrome (ACS) by sharing similar clinical and laboratory characteristics, making the differentiation between the two conditions very challenging. In both conditions, the patient presents with chest pain, ECG changes, elevated troponin, and wall motion abnormality by echocardiography [5,6,7]. The underlying mechanism of TTS is not fully understood, but several pathways, including catecholamine surge, epicardial coronary spasm, microvascular dysfunction, and genetic predisposition are involved in the pathogenesis of the disease [8,9,10,11]. Most patients are hemodynamically stable and exhibit complete echocardiographic recovery of the cardiac function; however, fulminant course with cardiogenic shock or intractable pulmonary edema may occur in rare cases [1,12]. Major complications include QT segment prolongation and ventricular arrhythmia, cardiogenic shock, dynamic mitral regurgitation secondary to left ventricular (LV) outflow tract obstruction, and systemic embolism such as stroke or transient ischemic attack [1]. In one study, male sex, reduced LV function on admission, and acute neurologic events were associated with less LV function recovery and subsequently with less favorable 1-year outcomes [13]. It is now well established that long-term prognosis of TTS is comparable to that of ACS [1]. In the current study, we aimed to compare the baseline characteristics and the clinical outcomes of hemodynamically stable TTS, with and without an emotional trigger before symptom onset.

## 2. Methods

### 2.1. Study Design and Population

In this retrospective cohort study, we included patients with ICD-9 diagnosis of takotsubo syndrome on discharge between 2017 and 2022 in the cardiology department at Galilee Medical Center. We included only cases of confirmed diagnosis of TTS. The diagnosis was confirmed finally by a senior cardiologist according to the appropriate criteria based on the clinical presentation, electrocardiographic changes, biomarkers, echocardiography, and coronary angiography or CCT showing no obstructive coronary disease. Based on history taking, we divided the cohort into two groups, patients with an obvious emotional trigger before symptom onset (with trigger), and patients with confirmed TTS without any identified trigger before hospitalization (without trigger).

### 2.2. Definitions

The revised Mayo Clinic diagnostic criteria are used when the diagnosis of TTS is suspected, based on the following:

1. Transient dyskinesia of the left ventricular midsegment.
2. Regional wall motion abnormality, beyond single coronary artery.
3. Absence of obstructive coronary artery disease or acute plaque rupture.
4. New electrocardiographic abnormalities or modest troponin elevation.
5. Absence of pheochromocytoma and myocarditis.

In most cases, coronary angiography is performed to rule out obstructive coronary artery disease, and a ventriculogram is performed for further confirmation of the diagnosis; however, cardiac computed tomography (CCT) may also be used. CCT may be preferred when another non-coronary condition is suspected, such as aortic dissection, or when the patient is not interested in invasive angiography.

### 2.3. Data Collection

Baseline characteristics, laboratory, and echocardiographic data were obtained based on the computerized files of the hospitalized patients. The presence or absence of an emotional trigger was clearly identified in all patients based on history taking (repeat focused history taking is usually performed after confirming the diagnosis). Electrocardiographic changes were reported in the case of the following findings: ST-segment elevation or depression, T-wave inversion, and QT segment prolongation. Maximal troponin, C reactive protein (CRP), and white blood cells (WBC) are presented. High sensitivity troponin I (Hs-TnI) level was measured using ARCHITECT assay (Abbott). Cut-off values for abnormal hs-TnI levels were above 20 ng/L and 30 ng/L for men and women, respectively. The patients were divided into two groups: with and without trigger based on the reported history. Triggers were also classified by negative, positive, or other (related to surgery or infection). Cases of “without trigger” were defined by ruling out any obvious trigger after comprehensive investigation. In all cases of TTS, a physician reevaluated the patients with a focus on identifying potential triggers.

### 2.4. Clinical Outcomes

Retrospectively, we included the following outcomes: length of stay of the index hospitalization, TTS-related complications (including atrial fibrillation, pulmonary congestion, and non-sustained ventricular tachycardia), 30-day recurrence, and 30-day death. Clinical outcomes were retrieved retrospectively based on the computerized files of the patients.

### 2.5. Statistical Analysis

Categorical variables are presented as percentages, while continuous variables are presented as median with interquartile range (IQR) or mean with standard deviation (SD). We used Fisher’s exact test and Chi square test to compare categorical variables between the two groups. Independent sample t-test or Mann–Whitney tests were used for continuous variables. The choice between those tests was made according to the distribution of the data; the Mann–Whitney test was used when a significant deviation from the normal distribution was found. To test the correlation between non-normal continuous variables we used Spearman’s test. All tests were conducted at a two-sided overall 5% significance level (*α* = 0.05). Multivariable logistic regression analysis and multivariable linear analysis were performed to examine the correlation between the trigger and complications and length of stay, respectively, adjusted to diabetes mellitus, neurological disease, and CRP. Odds ratio (OR) with 95% confidence interval (CI) were presented. To estimate time to recurrence, survival analysis was presented using the Kaplan–Meier method and Log-Rank test for the invariable analysis and Cox-regression with Hazard ratio (HR) and 95% CI for the multivariable analysis.

Statistical analysis was performed using R-IBM SPSS statistics (R-studio, V.4.0.3, Vienna, Austria). The study was approved by the local ethical committee of Galilee Medical Center.

## 3. Results

### 3.1. Baseline Characteristics

Among 86 patients with final diagnosis of TTS (93% female, mean age 68.8), 64 patients (74.4%) reported an emotional stress before admission. Table 1 summarizes the clinical characteristics and laboratory data of the study population.

IQR, interquartile range; CRP, C-reactive protein; DBP, diastolic blood pressure; ECG, electrocardiogram; Hs-TnI, high sensitivity troponin I; IQR, interquartile range; SBP, systolic blood pressure; SD, standard deviation; WBC, white blood cells.

Normal values: WBC (4.5–11.0); CRP (0.2–5.0); Hemoglobin (12.0–16.0); Hs-TnI (<20 for female, <30 for males); Creatinine (0.5–1.2).

The classification of the various triggers is provided in Table 2.

Examples of negative stress include grief or stressful arguments; stress related to work—the patient reported abrupt increase of required tasks; COVID-19, coronavirus disease-2019. Positive triggers may include a wedding, or a promotion at work

Overall, there was no statistical difference between groups, though some imbalance in proportions was present (e.g., for diabetes) (Table 3).

### 3.2. Clinical and Laboratory Parameters during the Index Hospitalization

During the index hospitalization, patients were monitored in the cardiology or cardiac care units. Hemodynamic and laboratory parameters are shown in Table 4. Of note, for patients in whom coronary angiography was not performed, coronary anatomy was demonstrated by cardiac computed tomography.

### 3.3. Outcomes

Overall, complications were reported in 27.9% of the study population (including atrial fibrillation, pulmonary congestion, and non-sustained ventricular tachycardia), with a trend for higher rates in the group with an emotional trigger, albeit it was not statistically significant (*p* = 0.069). Although QT-segment prolongation was documented in more than 50% of the patients, no events of Torsades de pointes were reported. The length of stay was significantly longer in the group with trigger before symptom onset. Beta-blockers and angiotensin converting enzyme inhibitors are often used when there is evidence of left ventricular dysfunction. Diuretic therapy was used in cases of volume overload. The outcomes during the index hospitalization and during 1 month after discharge are presented in Table 5.

In a multivariable regression model for the presence of trigger, diabetes mellitus (OR 0.63, 95% CI 0.2–1.96), neurological disease (OR 1.07, 95% CI 0.31–3.66), and CRP level (OR 1.0, 95% CI 0.99–1.01) were not associated with increased in-hospital complications. In linear regression adjusted to diabetes mellitus, neurological disease, and CRP level with the length of stay, only CRP was associated with increased hospital stay (*p* = 0.42, *p* = 0.64, and *p* = 0.003 respectively).

**Figure 1:**
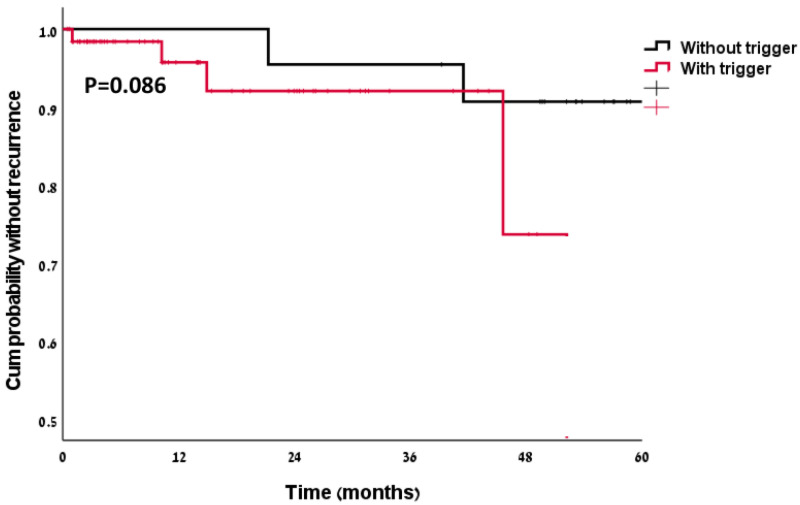
Kaplan–Meier curve for recurrence in the two groups.

In univariable survival analysis (Log-Rank, Mantel Cox), triggers were not associated with higher recurrence rate (*p* = 0.086), Similar results were obtained using the Cox regression model [HR 4.53, 95% CI 0.74–27.63, *p* = 0.101], also when the multivariable model was adjusted to diabetes, neurological disease, and CRP level. The Kaplan–Meier curve is presented in Figure 1.

### 3.4. Echocardiographic Follow Up

Forty-eight patients with reduced LV function (left ventricular ejection fraction (LVEF) < 50%) had repeated echocardiography after discharge. Of them, 41 (85.4%) experienced complete recovery of cardiac function (LVEF > 50%) within a range of 2–8 weeks, while a residual cardiac dysfunction was observed in 7 (14.6%) of them. Of note, all patients with residual cardiac dysfunction had an initial LVEF of 25% or less.

**Figure 2:**
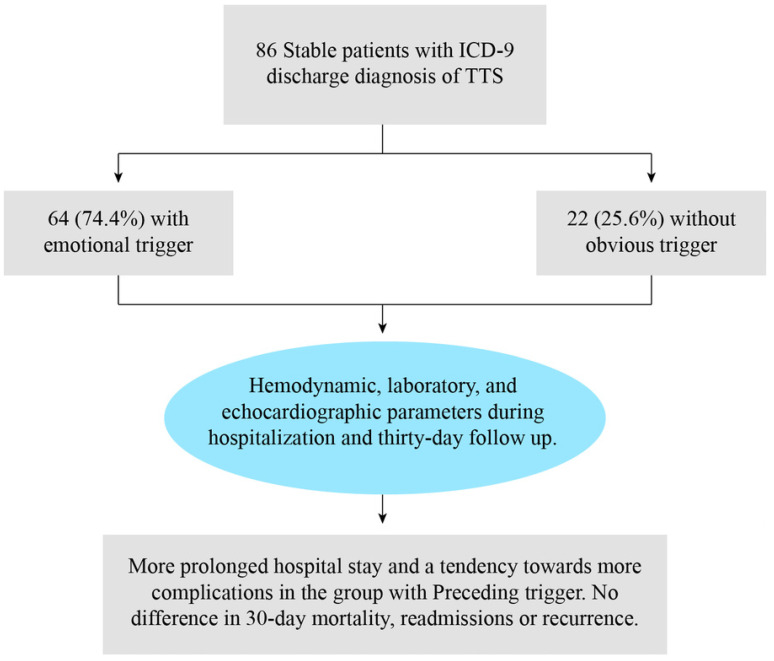
Flow diagram of the study.

The flow diagram of the study is illustrated in Figure 2.

## 4. Discussion

The role of stress in the pathogenesis of TTS is well established. In the current study, we aimed to compare the clinical outcomes of patients with TTS with and without a trigger before symptoms onset. The length of stay was longer in the group with a preceding trigger, probably driven by more prolonged symptoms. The impact of triggers on outcomes in TTS has been evaluated in previous studies, and a clear association was demonstrated between medical illness as the preceding trigger, and in-hospital mortality [2]. This association probably reflects the impact of the underlying disease on outcomes rather than the consequences of TTS. Some of the complications of TTS (such as arrhythmia and LVOT obstruction) may be explained by the abrupt surge of catecholamines during the acute phase of the disease [14,15,16,17,18]. Although there was no significant difference in the rate of complications between the two groups in the current study, this may be attributed to the small study population. We assume that all reported complications were related to TTS itself, because we included patients with stable hemodynamic and respiratory parameters without underlying severe illness during the index hospitalization, and without need for respiratory or circulatory mechanical support. TTS is mistakenly considered a benign condition, though the outcome is comparable to that of ACS. In the study by Templin et al., the mortality rate and major complications of patients with TTS were similar to those of patients with ACS [1]. Previous studies showed that TTS triggered by illness is associated with higher mortality rates in long-term follow up than acute coronary syndrome (ACS), while emotional stress-related TTS had better outcomes [2,19,20]. Thus, a new classification for prognostic purpose was proposed based on the preceding trigger [21]. It should be noted that, in all the patients in the study, the presence or absence of an emotional trigger was confirmed following a focused interview aimed at identifying possible triggers once the diagnosis of TTS had been suspected. We cannot claim that TTS with an emotional trigger represents a different disease with a different course and outcome because we could not eliminate all potential confounders. Therefore, we can rather conclude that TTS patients with a trigger may represent a different population from those presenting without a preceding trigger, and this may in part explain the difference in the clinical course. In our small study, the recurrence rate within five years was similar between the groups; however, large cohort studies are needed to address this issue. Overall, we found no correlation between the presence of an emotional trigger and short-term outcomes (30-day mortality or recurrence).

Despite the small sample size, we believe that our study provides some new insights.

First, we included only patients with emotional triggers before symptom onset and we excluded cases of TTS secondary to medical conditions that may mascaraed the course of TTS by dictating the outcome of the patient. Therefore, we tried to isolate the role of emotional stress in TTS and its implications on outcomes.

Second, we have provided 5-year outcomes. The relatively low recurrence rate that was observed (regardless of triggers) may strengthen other previous studies [22]. Further, studies including the measurement of catecholamines during the acute phase of different types of TTS (with and without trigger) are warranted.

## 5. Study Limitations

Our study has several limitations. First, the level of natriuretic peptide was not presented due to high missing values. Natriuretic peptide was shown in several studies as being a leading prognostic biomarker in TTS, in addition to its diagnostic role. Second, our small cohort exhibits a relatively stable course of the disease, with a relatively low rate of severe complications. Third, we do not have the long-term outcomes.

## 6. Conclusions

Patients with TTS presenting with an emotional trigger before symptoms onset may represent a different population from patients with TTS without a preceding trigger, characterized by a longer hospital stay without, however, a difference in 30-day mortality or recurrence rate.

## Figures and Tables

**Table 1:** Baseline clinical characteristics and laboratory data of the study population.

*n*	86
Age (mean + SD)	68.8 + 12.3
Female (*n*, %)	80 (93)
Preceding trigger (*n*, %)	64 (74.4)
Tobacco use (*n*, %)	18 (20.9)
Diabetes Mellitus (*n*, %)	24 (27.9)
Hypertension (*n*, %)	55 (64)
Hyperlipidemia (*n*, %)	49 (57)
Psychological disease (*n*, %)	12 (14)
Neurological disease (*n*, %)	15 (17.4)
ECG changes (*n*, %)	51 (59.3)
SBP (mmHg, mean ± SD)	122.6 ± 25.7
DBP (mmHg, mean ± SD)	74.0 ± 15.5
Heart rate (bpm, mean ± SD)	80.2 ± 17.7
O_2_ saturation (mean ± SD)	95.3 ± 5.3
WBC (×10^9^/L, mean ± SD)	10.5 ± 4.8
CRP [(mg/L), median, IQR]	17.4 (6.1, 40.1)
Hemoglobin (gr/dL, mean ± SD)	12.2 ± 1.6
Hs-TnI [(ng/L), median, IQR]	1922.5 (852, 5382.5)
Creatinine (mg/dL, mean ± SD)	0.9 ± 0.4

**Table 2:** Classification of the various triggers.

Negative Stress	48 (75.0%)
Positive stress	3 (4.7%)
Post-surgery	6 (9.4%)
Stress related to work	4 (6.3%)
COVID-19 related	3 (4.7%)

**Table 3:** Baseline characteristics in the two groups.

	with Trigger N = 64	without Trigger N = 22	*p* Value
Age (years)	68.3 ± 11.9	70.1 ± 13.6	0.56
Female %	59 (92.2)	21 (95.5)	1
Tobacco use (*n*, %)	13 (20.3)	5 (22.7)	0.77
Diabetes Mellitus (*n*, %)	21 (32.8)	3 (13.6)	0.07
Hypertension (*n*, %)	43 (67.2)	12 (54.5)	0.31
Hyperlipidemia (*n*, %)	38 (59.4)	11 (50.0)	0.47
Psychological disease (*n*, %)	9 (14.1)	3 (13.6)	1
Neurological disease (*n*, %)	14 (21.9)	1 (4.5)	0.056

**Table 4:** Hemodynamic and laboratory parameters in the two groups.

	with Trigger N = 64	without Trigger N = 22	*p* Value
ECG changes (%)	62.5	50	0.325
SBP (mmHg, mean ± SD)	122.3 ± 26.9	123.5 ± 22.2	0.881
DBP (mmHg, mean ± SD)	72.5 ± 16.1	78.5 ± 13.1	0.118
Heart rate (bpm, mean ± SD)	82.2 ± 18.3	74.6 ± 14.4	0.058
O_2_ saturation (mean ± SD)	95.2 ± 5.8	95.5 ± 3.3	0.624
WBC (×10^9^/L, mean ± SD)	10.7 ± 5.1	9.7 ± 3.7	0.793
CRP [(mg/L), median, IQR]	21.55 (7.93, 51.5)	9.55 (2.9, 25.25)	0.005
Hemoglobin (gr/dL, mean ± SD)	12.0 ± 1.6	12.9 ± 1.4	0.028
Hs-TnI [(ng/L), median, IQR]	2315 (889.75, 5790.5)	1232 (753.25, 4797.25)	0.252
Creatinine (mg/dL, mean ± SD)	0.92 ± 0.4	0.92 ± 0.4	0.939
LVEF (mean ± SD)	42 (38.5, 50.0)	40.5 (37.5, 51.25)	0.604

CRP, C-reactive protein; DBP, diastolic blood pressure; ECG, electrocardiogram; Hs-TnI, high sensitivity troponin I; IQR, interquartile range; LVEF, left ventricular ejection fraction; SBP, systolic blood pressure; SD, standard deviation; WBC, white blood cells. Normal values: WBC (4.5–11.0); CRP (0.2–5.0); Hemoglobin (12.0–16.0); Hs-TnI (<20 for female, <30 for males); Creatinine (0.5–1.2).

**Table 5:** Outcomes during the index hospitalization and 30-day mortality.

	with Trigger N = 64	without Trigger N = 22	*p*-Value
ICA (*n*, %)	51 (79.7)	21 (95.5)	0.074
Normal angiography (*n*, %)	40 (78.4)	18 (85.7)	0.47
Non-significant disease (*n*, %)	11 (21.6)	3 (14.3)	
Length of stay (days, mean ± SD)	4.28 + 1.98	3.0 + 1.38	0.002
Complications (*n*, %)	21 (32.8)	3 (13.6)	0.069
QT_C_ segment prolongation (*n*, %)	37 (57.8)	12 (54.5)	0.78
Use of medications			
Beta blockers (*n*, %)	19 (29.7)	7 (31.8)	0.85
ACE inhibitors (*n*, %)	10 (15.6)	3 (13.6)	0.82
Furosemide (*n*, %)	15 (23.4)	5 (22.7)	0.94
Inotropic support (*n*, %)	0	0	N/A
Mechanical ventilation	0	0	N/A
Recurrence within five years (*n*, %)	5 (7.8)	2 (9)	0.84
30-day mortality (%)	0	0	N/A
30-day readmission (%)	1	0	N/A

ACE, Angiotensin-converting enzyme; ICA, invasive coronary angiography; N/A not applicable; SD, standard deviation. Non-significant disease was defined as coronary artery stenosis ≤ 50%. Prolonged QTc was defined as >450 msec for men and >470 msec for women.

## Data Availability

The data supporting this study’s findings are available from the corresponding author upon reasonable request.
